# Quantifying paraspinal muscle tone and stiffness in young adults with chronic low back pain: a reliability study

**DOI:** 10.1038/s41598-018-32418-x

**Published:** 2018-09-25

**Authors:** Xiaoqian Hu, Di Lei, Le Li, Yan Leng, Qiuhua Yu, Xiaoyu Wei, Wai Leung Ambrose Lo

**Affiliations:** 1 Department of Rehabilitation Medicine, Guangdong Engineering and Technology Research Center for Rehabilitation Medicine and Translation, The First Affiliated Hospital, Sun Yat-sen University, Guangzhou, 510080 China; 2 Department of Electronic and Electrical Engineering, University College London, Torrington Place, London, WC1E 7JE UK

## Abstract

The reliability of a handheld myotonometer when used in a clinical setting to assess paraspinal muscle mechanical properties is unclear. This study aimed to investigate the between-session intra-rater reliability of a handheld myotonometer in young adults with low back pain (LBP) in a clinical environment. One assessor recorded lumbar paraspinal muscle tone and stiffness in an outpatient department on two occasions. The intraclass correlation coefficient (ICC), standard error of measurement (SEM), smallest real difference (SRD) and Bland-Altman analysis were conducted to assess reliability. The results indicated acceptable between-days intra-rater reliability (ICC > 0.75) for all measurements. The SEM of the muscle tone and stiffness measurements ranged between 0.20–0.66 Hz and 7.91–16.51 N/m, respectively. The SRD was 0.44–1.83 Hz for muscle tone and 21.93–52.87 N/m for muscle stiffness. SEM and SRD at L_1_-L_2_ were higher than those at other levels. The magnitude of agreement appeared to decrease as muscle tone and stiffness increased. The myotonometer demonstrated acceptable reliability when used in a clinical setting in young adults with chronic LBP. Measurements of the upper lumbar levels were not as reliable as those of the lower lumbar levels. The crural attachment of the diaphragm at L_1_ and L_2_ may affect paraspinal muscle tone and stiffness during respiratory cycles.

## Introduction

Chronic low back pain (LBP) is among the leading causes contributing to years of living with a disability worldwide^1^. The increasing prevalence of chronic LBP among adolescents and young adults is widely reported in the literature. A recent epidemiological study reported a prevalence rate of 42.4% per year among young adults^2^. Mechanical muscle properties such as muscle tone and stiffness are considered fundamental to muscle function and for maintaining energy efficient muscle contractions^3^. Altered tone and stiffness in the lumbar myofascial region has been identified in people with chronic LBP^4,5^ and may be related to underlying pathologies and symptoms^6^. Rehabilitation interventions such as manual therapy or therapeutic exercises are common techniques to manage chronic LBP due to their benefits in altering muscle tone and stiffness by reducing paraspinal muscle activity^7–9^. Paraspinal muscle tone and stiffness are often assessed clinically by palpatory techniques such as manual spinal stiffness assessment^10^ to guide treatment decisions and appraise treatment effectiveness^11^. However, the reliability of manual palpatory techniques has been repeatedly criticized^12,13^. Furthermore, advanced laboratory-based imaging studies such as diagnostic ultrasound^14^, magnetic resonance elastography^15^, ultrasonic shear wave elastography^16^ and electromyography are not always clinically feasible. Therefore, quantifying changes in paraspinal muscle tone and stiffness in a clinical setting continues to be a challenge.

The handheld myotonometer was developed as a mean of objectively quantifying mechanical muscle properties. The principle behind the myotonometer is to apply multiple short impulses over the muscle bulk via the testing probe to generate oscillations in the muscle fibres^17^. The oscillation waveform is reflective of the viscoelastic properties of the muscles. Published literature indicates that the device is reliable in assessing mechanical properties in a healthy population^18–21^ and in people with pathological conditions^22–30^ within a laboratory environment. Recent studies have also demonstrated the feasibility of using the handheld myotonometer to differentiate lumbar extensor fascia stiffness between young patients diagnosed with ankylosing spondylitis and healthy individuals^5,31^. Despite these positive results, several authors have raised doubts about the reliability of myotonometers when used in pathological groups^27^ or when used in a clinical environment^26^. This was given some support by two recently published studies that indicated varying degrees of reliability in different muscle groups and reduced reliability when operating the device in a clinical setting^25,26^. Other authors have also suggested that the operation of any handheld device may be influenced by the operator’s experience^32^, measuring technique^33^ and background noise of the clinical environment^27^. Therefore, the reliability found in one pathological population recorded in a laboratory setting is unlikely to be generalized to other pathological populations recorded in a clinical setting. In addition, in previous reliability studies, the test site on the skin surface was marked to minimize the confounding factor of site identification when the second measurement was taken. While leaving a mark on the skin surface may be feasible in an inpatient setting, it is not always possible in an outpatient setting due to the irregularity of outpatient appointments.

To date, we found no published data documenting the reliability of a handheld myotonometer in measuring paraspinal muscle tone and stiffness in young adults with chronic LBP in an outpatient setting. The reliability of the device when used in a clinical setting must first be established before it can be considered an outcome measure to monitor changes in paraspinal muscle tone and stiffness. Therefore, the aim of this study was to assess the between-session intra-rater reliability of a handheld myotonometer in young adults with chronic LBP in a musculoskeletal outpatient setting.

## Methods

### Study Setting

This single-centre study was conducted within the Rehabilitation Outpatient Department of The First Affiliated Hospital, Sun Yat-sen University. Measurements were taken while participants were lying prone on the assessment couch in a treatment cubicle of the musculoskeletal outpatient department. The assessor received three hours of training from a senior research physiotherapist who had extensive experience operating the device. The training included test site identification, the standard operating procedure of the device and supervised practice. The assessors then had one week of unsupervised practice with healthy individuals to familiarize themselves with the data collection protocol.

### Recruitment

Participants were recruited from the staff population at the local institute and students who were on clinical rotations at the local institute, using social media and internal announcements. Interested participants were asked to express their interest to a member of the research team. All potential participants were provided with an information sheet and encouraged to ask questions regarding the study. A member of the research team then conducted the screening to confirm eligibility.

### Sample population

The inclusion criteria were as follow: (1) age between 18 to 25, (2) persistent pain in the lumbar or lumbosacral region (between T12 and the gluteal fold) without radiation to the legs for at least 6 weeks prior to enrolment^34^, (3) did not receive intervention for at least four weeks prior to enrolment.

The exclusion criteria were as follow: (1) body mass index (BMI) >30 kg/m^2^, (2) scoliosis, 3) history of fracture or surgery in the pelvic or spinal areas, (4) history of neurological conditions, (5) pregnancy, (6) presence of other medical conditions other than chronic LBP, (7) presence of a wound in the lumbar spine region at the time of data collection.

### Ethics

The study was approved by the Medical Ethical Committee of the First Affiliated Hospital of Sun Yat-sen University [approval no: 2016(85)]. The study was conducted in accordance with the Declaration of Helsinki. An information sheet was provided to all participants. Written informed consent was obtained from all participants. The relevant guidelines and regulations of the local institute were strictly followed when conducting the study. Participants were informed that they could withdraw from the trial without giving a reason. All data set generated as part of the current study are available from the corresponding author upon reasonable request.

### Instrument

A handheld myotonometer (MyotonPRO^®^, Estonia) was used to quantify bilateral lumbar paraspinal muscle tone and stiffness. The testing probe of the myotonometer was placed vertical to the skin surface of the belly of the tested muscle. The probe was first loaded by pushing against the skin surface to the required depth. Once the required depth was reached (indicated by a change of indicator light from red to green), the device then applied three short impulses (one second apart) to induce damped oscillations within the muscle bulk. The oscillation pattern recorded by the transducer was used to calculate the mechanical muscle properties.

### Parameters

The parameters of muscle tone and stiffness at bilateral L_1_ to L_5_ levels were recorded. The device measures muscle tone as the natural oscillation frequency (Hz) which is calculated as Hz = 1/T, where T is the duration of oscillation measured in seconds. Muscle stiffness (N/m) is related to the maximal acceleration of oscillation and the deformation of the tissue recorded by the transducer^17^. The manufacturer of the handheld myotonometer indicated that the stiffness of tissues within 2 cm below the epidermis could be measured^31^. The depth of 2 cm is consistent with other models of soft tissue compliance metres^35^. The Oswestry Low Back Pain Disability Index (ODI)^36^ was used to assess the disability level related to back pain. Japanese Orthopedic Association Back Pain score (JOABP)^37^ was used to assess the multi-dimensional status of the disorder, including quality of life, pain intensity and level of disability. The numerical pain rating scale (NPRS) was used to record the level of pain (range between 0–10) that participants were experiencing at the time of data collection.

### Procedure

Demographic data including age, gender, height, weight and clinical information of LBP were recorded at the beginning of the data collection session. Participants were asked to recall their average level of pain over the previous 6 weeks. Parameters were recorded while participants lay prone with the lumbar region exposed. The test sites were identified using the method proposed in a previous study^5^. The assessor first located the highest level of the iliac crests to estimate the level between the spinous processes of L_3_ and L_4_. The spinous processes of L_1_ to L_5_ were then identified and marked. The test sites were marked as the extensor muscle bulk prominences that were on the same level as each of the lumbar spinous processes. Participants were asked to place their hands beside their head and to lie comfortably to achieve full relaxation. The study assumed that by lying in a prone position with the trunk relaxed, participant would be in their neutral lumbar lordosis position. Measurements were taken in the order of L_1_ to L_5_, starting from the left then progressing to the right. Participants were asked to hold their breath for five seconds at the end of inspiration to minimize the confounding factor resulting from changes of intra-abdomen pressure occurring with natural respiratory cycles. The complete procedure (including test site identification) was repeated by the same assessor on a second occasion, one week apart at a similar time. Data were removed from the device after the first measurement for purposes of blinding and to minimize memory bias effect.

### Data analysis

Statistical analyses were conducted using SPSS 20 software (IBM, Armonk, NY, US). The normality of muscle tone and stiffness data were assessed by the Kolmogorov-Smirnov test and frequency histograms. Sample population characteristics including age, gender, body mass index (BMI), NPRS, ODI and JOABP were assessed by descriptive statistics. The differences in tone and stiffness among lumbar levels were assessed by repeated measures ANOVA, followed by post hoc analysis with Bonferroni adjustment (adjusted critical value: p < 0.005). The between-days measurement differences in paraspinal muscle tone and stiffness were assessed by a paired t-test (p < 0.05). Relative intra-rater reliability was determined by the intraclass correlation coefficient (ICC) model 3, k. This study interpreted ICC levels as follows: Excellent >0.75, Good to Fair = 0.74–0.40, and Poor <0.40^38^. Absolute reliability was determined by the standard error of measurement (SEM)^39^ and the smallest real difference (SRD)^40^. Systematic bias between measurements was assessed by Bland-Altman plots and 95% limits of agreement (LOA)^41^.

### Ethical Approval and Consent to participate

The Medical Ethical Committee of the First Affiliated Hospital of Sun Yat-sen University reviewed and approved the present study [Ethics No. 2016(85)]. Informed written consent was obtained from all participants who took part in the present study.

## Results

### Demographics

Thirty participants with chronic LBP were recruited in the study. The characteristics of the sample population are presented in Table 1. Table 2 presents the clinical information of the sample population.

**Table 1:** A summary of the demographics of all participants.

Basic information	
Age (mean ± SD)	22 ± 2
Body Mass Index (mean ± SD)	21 ± 2.8
Gender, Female/Male	15/15
Dominant Side, Left/Right	1/29

**Table 2:** Clinical information of the chronic LBP cohort.

ODI (mean ± SD)	5 ± 2
NPRS (mean ± SD)	4 ± 1
JOABP (mean ± SD)	26 ± 6
Location of pain (left/ central/right)	7/19/4

### Muscle tone and stiffness at different lumbar levels

Repeated measures ANOVA indicated that there were significant differences in muscle tone and stiffness among different lumbar levels (p < 0.05). Post-hoc analysis with Bonferroni adjustment indicated the difference in muscle tone and stiffness between each lumbar level was significant, except for the muscle tone between L_1_-L_2_ on the right side.

### Between-days differences

The mean of the muscle tone and stiffness at each lumbar level recorded on the two occasions are presented in Table 3. Paired t-tests revealed that the between-days differences were not significant (*p* < 0.05) at all lumbar levels. No significant difference was observed between the left and right side pooled paraspinal muscle tone and stiffness.

**Table 3:** Results of the ICC analysis of the chronic LBP cohort.

Location		Variable	Mean(SD)			ICC (95% CI)
			1^st^ Measurement	2^nd^ Measurement	Mean of two measurements	
Left Side	L1	Frequency (Hz)	16.5 (2.31)	16.4 (1.79)	16.5	0.88 (0.75–0.94)
		Stiffness (N/m)	345.1 (84.94)	341.4 (78.72)	343.3	0.92 (0.84–0.96)
	L2	Frequency (Hz)	16.0 (1.93)	16.0 (1.74)	16	0.88 (0.76–0.94)
		Stiffness (N/m)	332.7 (91.05)	325.3 (83.55)	329	0.90 (0.79–0.95)
	L3	Frequency (Hz)	15.6 (1.77)	15.5 (1.82)	15.6	0.94 (0.87–0.97)
		Stiffness (N/m)	311.8 (87.61)	302.5 (87.51)	307.2	0.93 (0.85–0.97)
	L4	Frequency (Hz)	15.8 (1.98)2	15.1 (2.07)	15.5	0.96 (0.92–0.98)
		Stiffness (N/m)	285.2 (86.61)	276.3 (86.00)	280.9	0.95 (0.90–0.99)
	L5	Frequency (Hz)	14.8 (2.26)	14.6 (2.32)	14.7	0.96 (0.91–0.98)
		Stiffness (N/m)	257.6 (93.45)	247.7 (95.39)	252.7	0.95 (0.89–0.98)
Right Side	L1	Frequency (Hz)	16.3 (1.77)	16.7 (1.95)	16.5	0.81 (0.59–0.91)
		Stiffness (N/m)	343.9 (68.81)	349.5 (73.51)	346.7	0.90 (0.79–0.95)
	L2	Frequency (Hz)	16.1 (1.57)	16.5 (1.78)	16.3	0.85 (0.68–0.98)
		Stiffness(N/m)	332.0 (71.28)	339.1 (76.28)	335.6	0.86 (0.71–0.94)
	L3	Frequency (Hz)	15.6 (1.68)	16.0 (1.86)	15.8	0.93 (0.85–0.97)
		Stiffness (N/m)	307.8 (81.10)	319.4 (83.69)	313.6	0.94 (0.87–0.97)
	L4	Frequency (Hz)	15.2 (2.00)	15.3 (2.06)	15.3	0.96 (0.92–0.98)
		Stiffness (N/m)	287.9 (86.97)	291.5 (86.02)	289.7	0.95 (0.90–0.98)
	L5	Frequency (Hz)	14.8 (261.70)	14.8 (2.28)	14.8	0.96 (0.91–0.98)
		Stiffness (N/m)	261.7 (94.51)	261.4 (92.08)	261.6	0.96 (0.91–0.99)

### Intraclass correlation coefficient

The ICCs of all parameters at each lumbar level range between 0.81 to 0.96, indicating excellent between-days intra-rater reliability. Detailed results of the ICC analysis are presented in Table 3. The ICC of the pooled muscle tone on the left was 0.93 (CI: 0.91–0.95) and 0.92 (CI: 0.88–0.94) on the right. For pooled muscle stiffness, the ICC was 0.94 (CI: 0.92–0.96) on the left and right side.

### SEM and SRD

The SEM for all muscle tone measurements ranged between 0.2–0.7 Hz. The SEM for all muscle stiffness measurements ranged between 7.9–16.5 N/m. The SRD for all muscle tone measurements ranged between 0.4–1.8 Hz. The SRD for all muscle stiffness ranged between 21.9–52.9 N/m. Table 4 illustrates the SEM and SRD of the muscle tone and stiffness recorded at each level.

**Table 4:** Results of absolute reliability indices of the chronic LBP cohort.

		Parameters	SEM	SRD	95% LOA	
					Lower	Upper
Left Side	L1	Frequency(Hz)	0.5	1.3	−2.6	2.7
		Stiffness(N/m)	12.3	34.1	−82.6	89.9
	L2	Frequency(Hz)	0.4	1.1	−2.3	2.4
		Stiffness(N/m)	16.5	45.8	−95.5	110.2
	L3	Frequency(Hz)	0.2	0.6	−1.6	1.8
		Stiffness(N/m)	12.2	33.7	−79.6	98.1
	L4	Frequency(Hz)	0.2	0.4	−1.4	1. 7
		Stiffness(N/m)	7.9	21.9	−62.6	80.5
	L5	Frequency(Hz)	0.2	0.5	−1.6	2.1
		Stiffness(N/m)	9.6	26.6	−72.7	92.5
Right Side	L1	Frequency(Hz)	0.7	1.8	−3.3	2.6
		Stiffness(N/m)	13.5	37.3	−89.5	78.2
	L2	Frequency(Hz)	0.5	1.3	−2.7	2.1
		Stiffness(N/m)	19.1	52.9	−107.8	93.5
	L3	Frequency(Hz)	0.3	0.70	−2.2	1.5
		Stiffness(N/m)	10.1	27.9	−90.0	66.9
	L4	Frequency(Hz)	0.2	0.4	−1.7	2.0
		Stiffness(N/m)	8.3	23.0	−77.0	69.9
	L5	Frequency(Hz)	0.2	0.6	−1.9	1.9
		Stiffness(N/m)	8.2	22.8	−75.8	76.3

### Bland-Altman analysis

**Figure 1:**
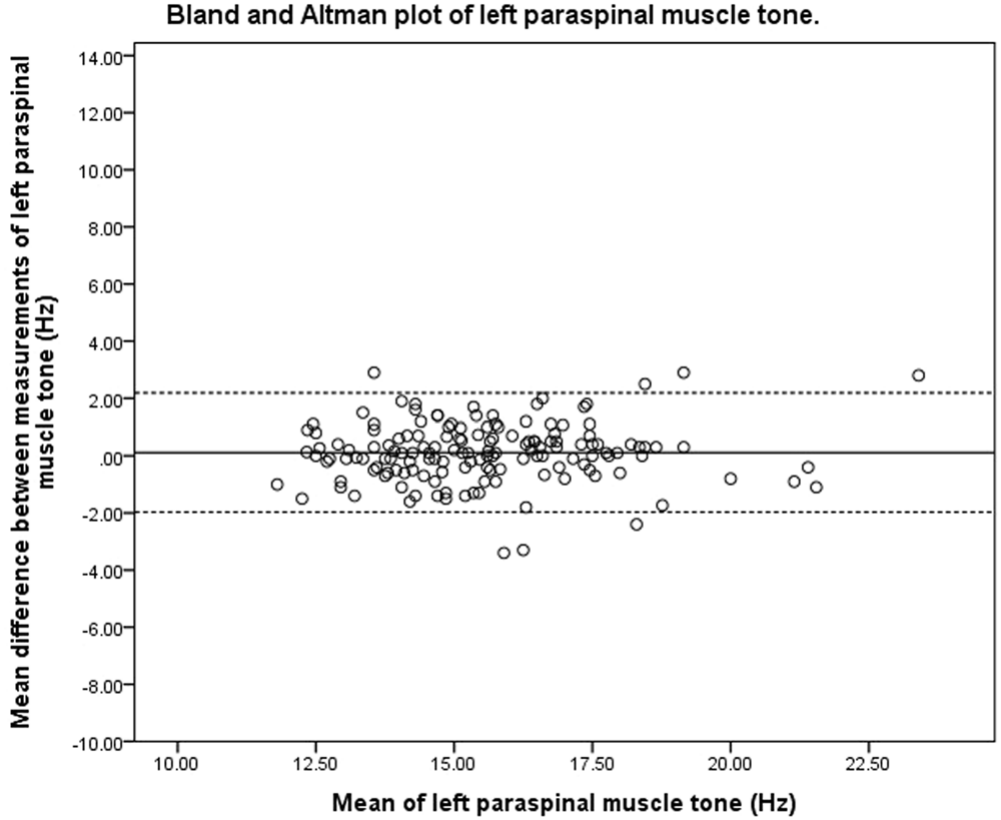
Bland and Altman plot of pooled left paraspinal muscle tone.

**Figure 2:**
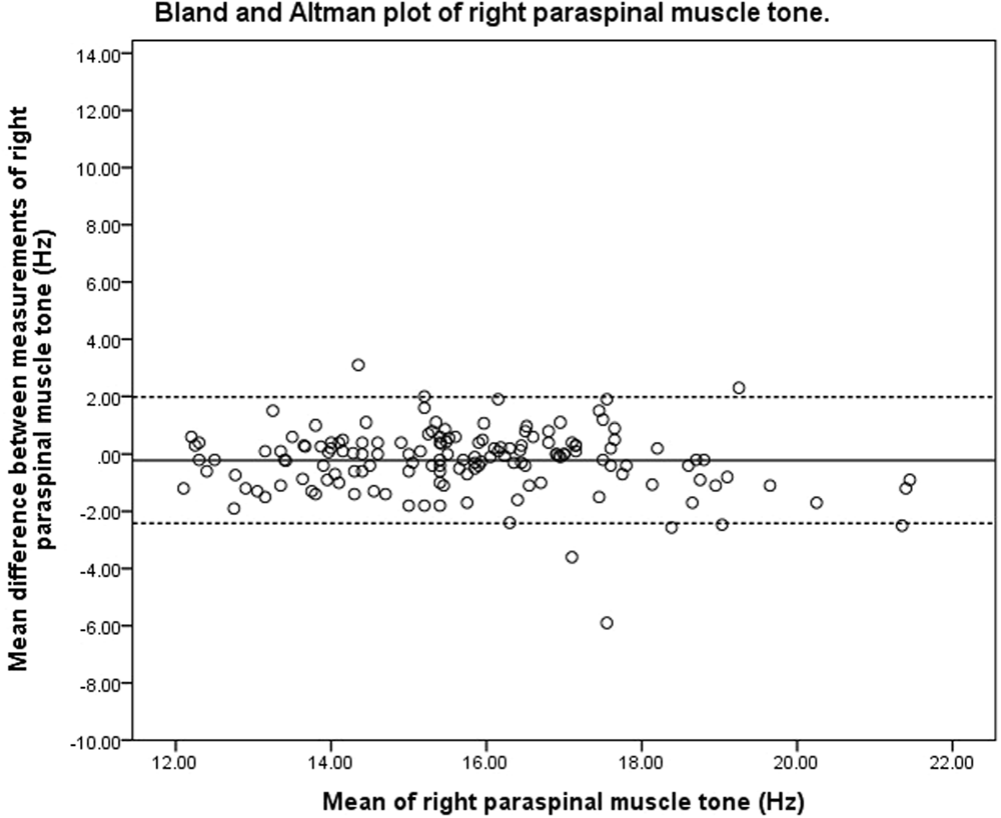
Bland and Altman plot of pooled right paraspinal muscle tone.

**Figure 3:**
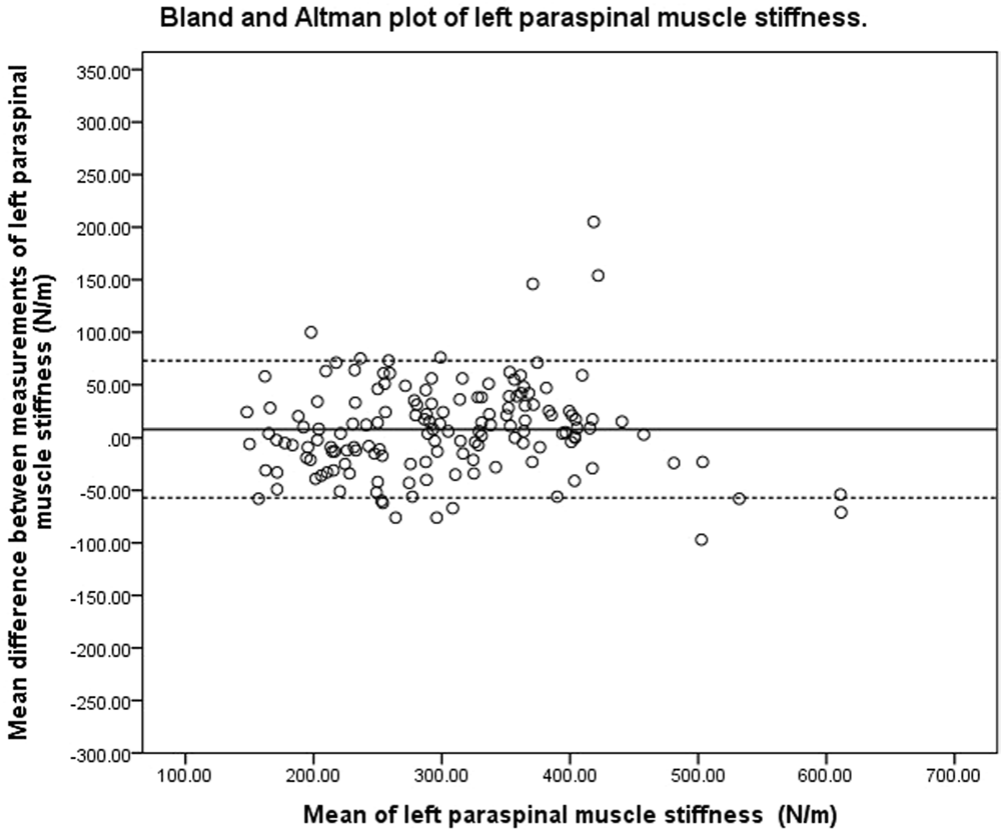
Bland and Altman plot of pooled left paraspinal muscle stiffness.

**Figure 4:**
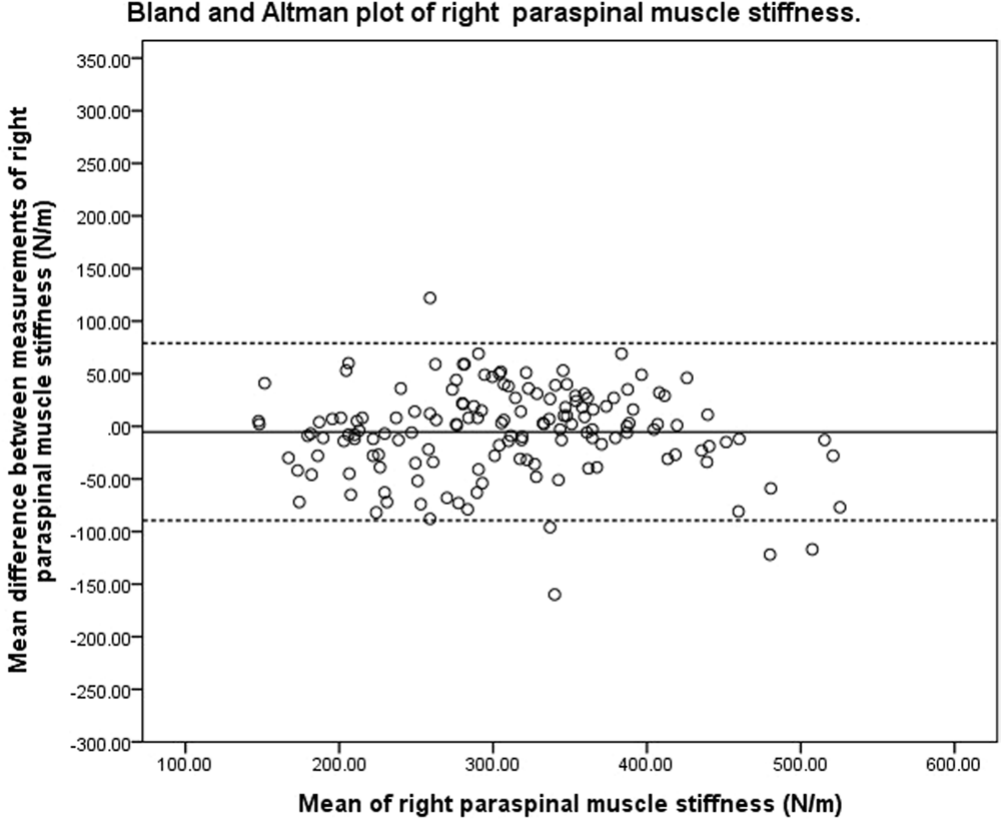
Bland and Altman plot of pooled right paraspinal muscle stiffness.

The 95% LOA of pooled muscle tone on the left and right side were between −2.0 to 2.1 Hz and −2.4 to 2.0 Hz, respectively. For pooled muscle stiffness, the 95% LOA on the left and right side were between −79.8 to 94.7 N/m and between −89.5 to 79.0 N/m, respectively. Bland-Altman plots (Figs 1–4) indicated no systematic bias between the two measurements. However, the magnitude of agreement appeared to decrease when paraspinal muscle tone and stiffness increased.

## Discussion

This study is among the first to assess the reliability of a handheld myotonometer when used in a musculoskeletal outpatient setting to quantify paraspinal muscle tone and stiffness in young adults with chronic LBP. The results indicated acceptable between-day intra-rater reliability. The errors between measurements were small with no systematic bias.

### Paraspinal muscle mechanical properties

The present study quantified paraspinal muscle tone and stiffness measurements in young adults with chronic LBP at different spinal levels. The results indicated a decrease in muscle tone and stiffness from L_1_ to L_5_. The decreasing values may be related to the anatomy of paraspinal muscles that run more inferiorly towards lower lumbar levels. Thus, the measurements taken at the lower lumbar levels may reflect greater contribution from superficial soft tissue than those at the upper levels. This outcome is supported by a previous study of MRI images that indicated, in the absence of spinal oedema, that the soft tissue above the paraspinal muscles in adults age between 22–81 years old was thicker at L_5_ than that at L_1_^42^. If such a finding also applies to the sample population of the current study, it would provide a possible explanation for the decreasing muscle tone and stiffness from L_1_ to L_5_. Another possible contributing factor for the decrease in muscle tone and stiffness at the lower lumbar level is the effect of the sequential measurement method from L_1_ to L_5_. Sequential measurement refers to the data collection sequence from L_1_ to L_5_ beginning on the left side then progressing to the right. It is currently unknown if this type of sequential measurement is likely to affect muscle properties, since the majority of published studies about lumbar paraspinal muscle stiffness only record measurements taken at one particular level.

### Intraclass correlation coefficient

The ICC index reflects the degree of consistency and agreement between the two ratings^43^. The higher the agreement between measurements, the closer the value is to 1. Some authors have suggested that the existing interpretation of ICC is mostly based on data from inter-rater reliability, and a higher ICC value should be expected for intra-rater reliability^44^, potentially reaching 0.8 or above to be considered acceptable^25^. In the present study, the ICCs for the paraspinal spinal muscle tone and stiffness at each lumbar level were above 0.8, indicating acceptable intra-rater reliability. The observed ICC values were consistent with a recently published study that investigated the within session intra-rater reliability of paraspinal muscle stiffness at L_4_ level (ICC = 0.99)^45^ at rest. The lower ICC score observed in this study may be related to a different data collection process. In the study by Kelly *et al*., the interval between measurements was not documented and repeated measurements appeared to be taken in close succession, using the test site marked during the first recording. Findings in this study suggest that the handheld myotonometer may be a reliable way to quantify muscle stiffness in a clinical setting. The ICCs of the muscle tone observed in this study are consistent with those reported in a study that investigated the between-day intra-rater reliability of muscle tone of limb muscles in a clinical setting (ICCs range between 0.75–0.82, CI range between 0.37–0.93)^25^. As with several other published reliability studies concerning handheld myotonometers in clinical^25,26^ and laboratory settings^18,19,46,47^, the second measurements were taken at the location that was marked during the first data collection session. Therefore, the majority of existing studies do not take into consideration a potential error related to site identification. The high ICCs observed in this study indicated that the reliability of quantifying paraspinal muscle tone were unlikely to be affected by the site identification process. Despite high ICC values observed in individual lumbar levels and pooled data, the interpretation of ICC was not straightforward since there was minimal clinical data that would suggest whether the observed reliability levels were clinically acceptable. In addition, the lower bound of 95% CI recorded at L_1_ (tone) and L_2_ (tone and stiffness) on the right side were below the acceptable level of 0.75 previously proposed^48^. The wide CIs implied low power due to the small sample size. Thus, no firm conclusion can be drawn from the ICC analysis.

### SEM and SRD

The SEM and SRD are absolute indices that reflect the reliability of an instrument. SEM refers to the estimation of how repeated measures tend to be distributed around the “true” score. SRD refers to the smallest value that could be interpreted as “real” change. Any observed change that is above the SRD indicates the change is unlikely due to measurement error. The smaller the SEM and SRD values, the higher the reliability of the instrument. Insufficient data are currently available for direct comparisons of the SEM and SRD values of paraspinal muscles. The SEM observed in this study was less than 0.7 Hz for muscle tone and 20 N/m for muscle stiffness. The small SEM observed in this study was consistent with a study that previously investigated the between-day reliability of peripheral muscle tone in a clinical setting within the stroke population. Lo *et al*.^25^ reported the SEM values of the biceps brachii and rectus femoris of 0.76 Hz and 0.83 Hz, respectively. The SEM of triceps muscle tone (0.70 Hz) recorded from a stroke population in a laboratory setting was also consistent with the SEM observed in the present study^23^. The SEM and SRD of muscle tone and stiffness recorded at bilateral L_1_ and L_2_ levels had higher values compared to L_3_ to L_5_ levels, indicating higher variations around that “true” score on repeated measurements and requiring larger differences to be considered real change. This was consistent with previous studies in which the myotonometer device had different reliability when measuring different muscle groups. The difference in the reduced reliability observed at the upper lumbar levels may be related to the change in spinal stiffness throughout the respiratory cycle. The crural diaphragm attachment extends to the transverse process of L_2_ and, therefore, contraction of the diaphragm has a direct effect on spinal stiffness^49^. A previous study provided evidence to support that L_4_ stiffness does not change with lung volumes when breathing within a normal tidal range, whereas L_2_ stiffness increases at all increments in lung volume^50^. The present study attempted to minimize the effect of respiration by taking the measurements at end tidal inspiration. However, end tidal inspiration volume was not objectively quantified, and it could not be confirmed that participants inspired the same volume on the two occasions. The difference in inspiration volume may have affected the muscle properties at L_1_ and L_2_, which in turn would influence the reliability of the reading.

### Bland-Altman analysis

The purpose of Bland-Altman analysis is to identify systematic bias and the magnitude of disagreement between measures. The Bland-Altman plots did not indicate systematic bias between the two measurements, though the magnitude of disagreement appeared to increase as tone and stiffness increased. This finding is consistent with a published study that investigated the reliability of measuring the mechanical properties of biceps brachii in the elderly with and without paratonia^27^. The study similarly reported reduced reliability as muscle tone increased. The range of 95% LOA observed at the bilateral upper lumbar vertebrae was wider than that observed at the lower lumbar vertebrae. The variation in consistency was similar to the findings of the ICC, SEM and SRD indices of the present study. One of the difficulties in interpreting 95% LOA was the lack of a universal clinically accepted range. In a study that previously investigated the difference in paraspinal muscle stiffness in young adults with back pain resulting from ankylosing spondylitis^31^, a difference of 30 N/m in muscle stiffness was noted at baseline between the back-pain group and healthy controls. This difference was larger than the SRD and was within the 95% LOA observed in the present study. These findings were indicative of handheld myotonometer’s potential to quantify mechanical muscle properties in a clinical setting. There has been no study investigating intervention-induced quantitative changes of muscle tone and stiffness measured by myotonometer in the chronic LBP population. Therefore, there is currently insufficient published data to indicate if the observed range of error in the present study is clinically acceptable. The findings of the present study thus provide a reference for measuring changes of paraspinal muscle tone and stiffness on different days.

## Limitations

The lack of other objective measures to ensure the muscles were at a comparable state during the two recording sessions may contribute to the underestimation of reliability. Although participants were advised to refrain from physical exercises on the day of data collection, the amount of physical activities on that day could not be controlled. However, as the study protocol closely mimicked clinical routine practice, it is not always possible to control the physical activities of people who attend outpatient appointments.

Lumbar lordosis was not strictly controlled within the study and the degree of lumbar extension or flexion may affect the reliability reading. However, controlling lumbar lordosis by means such as strapping the participant to the examination plinth may affect the relaxation state and subsequently muscle properties. In addition, controlling lumbar lordosis may lead to the confounding factor of repositioning lumbar lordosis at different measurement time points. Asking the participant to lie prone with the body relaxed is a common clinical practice and frequently cited method in published literature assessing lumbar spinal muscle function.

Because this study did not test the reliability of the device on a range of participants with different levels of muscle tone and stiffness, the findings may not be the generalizable. There is also the limitation of myotonometer technology itself, which measures not only the properties of a particular muscle structure but also those of the soft tissue above the muscle fibre. Thus, the indirect nature of the measuring technique might generate false measurements, since the “true” value of muscle properties may be masked by the stiffer fascia located superficially to the paraspinal muscle. However, a previous study indicated that the stiffness of the erector spinae at rest measured by myotonometer was moderately correlated with muscle stiffness measured by elastography. Changes in erector spinae stiffness measured by a myotonometer at different contraction intensities were also comparable with stiffness measured by elastography^45^. Another study suggested that the surface electromyography activity is concurrent with the extensor myofascial tone^5^, though other authors stated it was unlikely that the deeper multifidus would be measured. However, there is no empirical evidence that indicates whether the indentation force might affect structures below the erector spinae. Despite the limitation of the technology, it should not impact the reliability analysis since the readings were compared between the two measurements, rather than between different lumbar levels. Further investigation is recommended to understand the exact spinal tissue that is probed by the myotonometer in order to improve the clinical application of the device.

This study analysed the data based on the left and right side of the spine rather than on the pain location. We acknowledge that this comparison may hide important information relative to the pain side. However, as the study was not primarily aimed to assess the difference in muscle properties between the pain and non-pain side, the study included small and an unequal number of participants with unilateral pain. This comparison between pain and non-pain side is therefore unlikely to be statistically meaningful.

## Conclusions

The present study demonstrated acceptable between-days intra-rater reliability when using a myotonometer to measure muscle tone and stiffness in young adults with chronic LBP in an outpatient setting. The agreement between measurements is acceptable. The error range at L3 to L5 levels is consistent with existing literature. The error range recorded at L1 and L2 indicates that a larger change is required to be deemed a real change in muscle tone and stiffness.

## References

[1] Global Burden of Disease Study, C (2015). Global, regional, and national incidence, prevalence, and years lived with disability for 301 acute and chronic diseases and injuries in 188 countries, 1990–2013: a systematic analysis for the Global Burden of Disease Study 2013. Lancet.

[2] Ganesan S, Acharya AS, Chauhan R, Acharya S (2017). Prevalence and Risk Factors for Low Back Pain in 1,355 Young Adults: A Cross-Sectional Study. Asian spine journal.

[3] Masi AT, Hannon JC (2008). Human resting muscle tone (HRMT): narrative introduction and modern concepts. J Bodyw Mov Ther.

[4] Haladaj R, Topol M (2016). Multiple Impulse Therapy in the Assessment of Paraspinal Muscle Tone in Patients with Low BackPain. Ortopedia, traumatologia, rehabilitacja.

[5] Nair K (2016). Stiffness of resting lumbar myofascia in healthy young subjects quantified using a handheld myotonometer and concurrently with surface electromyography monitoring. Journal of bodywork and movement therapies.

[6] Kawchuk GN (2001). The diagnostic performance of vertebral displacement measurements derived from ultrasonic indentation in an *in vivo* model of degenerative disc disease. Spine (Phila Pa 1976).

[7] Fryer G, Morse CM, Johnson JC (2009). Spinal and sacroiliac assessment and treatment techniques used by osteopathic physicians in the United States. Osteopathic medicine and primary care.

[8] Lehman G (2012). Kinesiological research: the use of surface electromyography for assessing the effects of spinal manipulation. J Electromyogr Kinesiol.

[9] Miller EM, Bazrgari B, Nussbaum MA, Madigan ML (2013). Effects of exercise-induced low back pain on intrinsic trunk stiffness and paraspinal muscle reflexes. Journal of biomechanics.

[10] Masaki M (2017). Association of low back pain with muscle stiffness and muscle mass of the lumbar back muscles, and sagittal spinal alignment in young and middle-aged medical workers. Clinical biomechanics (Bristol, Avon).

[11] Abbott JH (2009). Manual physical assessment of spinal segmental motion: intent and validity. Man Ther.

[12] Seffinger MA (2004). Reliability of spinal palpation for diagnosis of back and neck pain: a systematic review of the literature. Spine (Phila Pa 1976).

[13] Jonsson A, Rasmussen-Barr E (2018). Intra- and inter-rater reliability of movement and palpation tests in patients with neck pain: A systematic review. Physiotherapy theory and practice.

[14] Muraoka T, Chino K, Muramatsu T, Fukunaga T, Kanehisa H (2005). *In vivo* passive mechanical properties of the human gastrocnemius muscle belly. J Biomech.

[15] Dresner MA (2001). Magnetic resonance elastography of skeletal muscle. J Magn Reson Imaging.

[16] Qiu W, Wang C, Xiao Y, Qian M, Zheng H (2015). A new shear wave imaging system for ultrasound elastography. Conference proceedings:… Annual International Conference of the IEEE Engineering in Medicine and Biology Society. IEEE Engineering in Medicine and Biology Society. Annual Conference.

[17] Gapeyeva, H. & Vain, A. *Methodological guide: principles of applying Myoton in physical medicine and rehabilitation*. (Tartu, Estonia: Muomeetria Ltd, 2008).

[18] Aird L, Samuel D, Stokes M (2012). Quadriceps muscle tone, elasticity and stiffness in older males: Reliability and symmetry using the MyotonPRO. Archives of gerontology and geriatrics.

[19] Bailey L, Samuel D, Warner MB, Stokes M (2013). Parameters representing muscle tone, elasticity and stiffness of biceps brachii in healthy older males: symmetry and within-session reliability using the MyotonPRO. Journal of Neurological Disorders.

[20] Mooney, K., Warner, M. B. & Stokes, M. Symmetry and within-session reliability of mechanical properties of biceps brachii muscles in healthy young adult males using the MyotonPRO devicereliability of muscle tone, stiffness and elasticity measurements of rectus femoris and bicepsbrachii in healthy young and older males. *Working Papers in Health Sciences* (2013).

[21] Davidson Melissa J., Bryant Adam L., Bower Wendy F., Frawley Helena C. (2017). Myotonometry Reliably Measures Muscle Stiffness in the Thenar and Perineal Muscles. Physiotherapy Canada.

[22] Jarocka E, Marusiak J, Kumorek M, Jaskolska A, Jaskolski A (2012). Muscle stiffness at different force levels measured with two myotonometric devices. Physiol Meas.

[23] Chuang LL (2013). Relative and absolute reliabilities of the myotonometric measurements of hemiparetic arms in patients with stroke. Archives of physical medicine and rehabilitation.

[24] Chuang LL, Wu CY, Lin KC, Lur SY (2012). Quantitative mechanical properties of the relaxed biceps and triceps brachii muscles in patients with subacute stroke: a reliability study of the myoton-3 myometer. Stroke research and treatment.

[25] Lo WLA (2017). Between-days intra-rater reliability with a hand held myotonometer to quantify muscle tone in the acute stroke population. Sci Rep.

[26] Lo WLA, Zhao JL, Li L, Mao YR, Huang DF (2017). Relative and Absolute Interrater Reliabilities of a Hand-Held Myotonometer to Quantify Mechanical Muscle Properties in Patients with Acute Stroke in an Inpatient Ward. BioMed research international.

[27] Van Deun, B. *et al*. Reproducible Measurements of Muscle Characteristics Using the MyotonPRO Device: Comparison Between Individuals With and Without Paratonia. *Journal of geriatric physical therapy (2001*), 10.1519/jpt.0000000000000119 (2016). 10.1519/JPT.0000000000000119 28005829

[28] Li X, Shin H, Li S, Zhou P (2017). Assessing muscle spasticity with Myotonometric and passive stretch measurements: validity of the Myotonometer. Scientific Reports.

[29] Frohlich-Zwahlen AK, Casartelli NC, Item-Glatthorn JF, Maffiuletti NA (2014). Validity of resting myotonometric assessment of lower extremity muscles in chronic stroke patients with limited hypertonia: a preliminary study. Journal of electromyography and kinesiology: official journal of the International Society of Electrophysiological Kinesiology.

[30] Chuang LL, Wu CY, Lin KC (2012). Reliability, validity, and responsiveness of myotonometric measurement of muscle tone, elasticity, and stiffness in patients with stroke. Archives of physical medicine and rehabilitation.

[31] Andonian BJ (2015). Greater Resting Lumbar Extensor Myofascial Stiffness in Younger Ankylosing Spondylitis Patients Than Age-Comparable Healthy Volunteers Quantified by Myotonometry. Archives of physical medicine and rehabilitation.

[32] Kim SG, Kim EK (2016). Test-retest reliability of an active range of motion test for the shoulder and hip joints by unskilled examiners using a manual goniometer. Journal of physical therapy science.

[33] Farooq MN, Mohseni Bandpei MA, Ali M, Khan GA (2016). Reliability of the universal goniometer for assessing active cervical range of motion in asymptomatic healthy persons. Pak J Med Sci.

[34] O’Sullivan P (2005). Diagnosis and classification of chronic low back pain disorders: maladaptive movement and motor control impairments as underlying mechanism. Manual Therapy.

[35] Arokoski JP, Surakka J, Ojala T, Kolari P, Jurvelin JS (2005). Feasibility of the use of a novel soft tissue stiffness meter. Physiological measurement.

[36] Liu Q, Mai M, Xiao LA (2010). Responsiveness of Chinese version of Oswestry disability index in subjects with chronic low back pain. Chinese Journal of Rehabilitation Medicine.

[37] Yao M (2018). Simplified Chinese Version of the Japanese Orthopaedic Association Back Pain Evaluation Questionnaire: Cross-cultural Adaptation, Reliability, and Validity for Patients With Low Back Pain. Spine (Phila Pa 1976).

[38] Portney, L. G. & Watkins, M. P. Foundations of Clinical Research: Application to Practice (2008).

[39] Hopkins WG (2000). Measures of reliability in sports medicine and science. Sports medicine (Auckland, N.Z.).

[40] Rankin G, Stokes M (1998). Reliability of assessment tools in rehabilitation: an illustration of appropriate statistical analyses. Clin Rehabil.

[41] Bland JM, Altman DG (1986). Statistical methods for assessing agreement between two methods of clinical measurement. Lancet (London, England).

[42] West W, Brady-West D, West KP (2018). A comparison of statistical associations between oedema in the lumbar fat on MRI, BMI and Back Fat Thickness (BFT). Heliyon.

[43] Bruton A, Conway JH, Holgate ST (2000). Reliability: What is it, and how is it measured?. Physiotherapy.

[44] To T, Estrabillo E, Wang C, Cicutto L (2008). Examining intra-rater and inter-rater response agreement: a medical chart abstraction study of a community-based asthma care program. BMC medical research methodology.

[45] Kelly JP (2018). Characterization of tissue stiffness of the infraspinatus, erector spinae, and gastrocnemius muscle using ultrasound shear wave elastography and superficial mechanical deformation. Journal of Electromyography and Kinesiology.

[46] Agyapong-Badu S, Warner M, Samuel D, Stokes M (2016). Measurement of ageing effects on muscle tone and mechanical properties of rectus femoris and biceps brachii in healthy males and females using a novel hand-held myometric device. Archives of gerontology and geriatrics.

[47] Bizzini M, Mannion AF (2003). Reliability of a new, hand-held device for assessing skeletal muscle stiffness. Clin Biomech (Bristol, Avon).

[48] Lee J, Koh D, Ong CN (1989). Statistical evaluation of agreement between two methods for measuring a quantitative variable. Computers in biology and medicine.

[49] Standring, S. *et al*. *Gray’s anatomy. The anotomical basis of clinical practice*. (Churchill Livingstone, Elsevier., 2008).

[50] Shirley D, Hodges PW, Eriksson AE, Gandevia SC (2003). Spinal stiffness changes throughout the respiratory cycle. Journal of applied physiology (Bethesda, Md.: 1985).

